# Impacts of industrial agglomeration on the energy consumption structure’s low-carbon transition process: A spatial and nonlinear perspective

**DOI:** 10.1371/journal.pone.0307893

**Published:** 2024-09-06

**Authors:** Yuqing Liu

**Affiliations:** Department of Business, Zhengzhou University, Zhengzhou, China; University of Regina, CANADA

## Abstract

Based on panel data collected from 2003 to 2020 across 30 provinces in China, the paper employs the spatial vector angle method and spatial Durbin model to investigate industrial agglomeration’s nonlinear and spatial spillover effects on the energy consumption structure’s low-carbon transition process (Lct). The results indicate the following: First, the influence of industrial agglomeration on Lct exhibits an inverted U-shaped pattern. As the degree of industrial agglomeration expands, its effect on Lct shifts from positive to negative. Second, industrial agglomeration demonstrates spatial spillover effects. It promotes the improvement of Lct in neighboring provinces through agglomeration effects. However, the continuous expansion of industrial agglomeration inhibits the improvement of Lct in neighboring provinces through congestion effects. Third, the heterogeneity test finds that industrial agglomeration has a significant role in promoting Lct in the samples of eastern region, but this effect is not significant in the samples of western and middle regions.

## 1. Introduction

Since the initiation of China’s reform and opening-up program, the nation has experienced remarkable economic growth, with its GDP soaring from 367.87 billion to 113,323.98 billion yuan between 1978 and 2021, reflecting an impressive annual growth rate of 14.25 percent. However, this growth has come at the cost of societal issues, notably excessive energy use and environmental pollution, arising from a historically haphazard development approach. China is steering its economy from fast growth to a high-quality growth model, emphasizing the simultaneous pursuit of economic prosperity and environmental sustainability. This transition aligns with the imperative outlined in the 19th CPC National Congress report, stressing the need for a contemporary energy system characterized by cleanliness, low-carbon footprint, safety, and efficiency. Crucial to fostering China’s energy revolution and urgent economic and social transformation is realizing clean, low-carbon development and optimizing the energy consumption structure. These endeavors are fundamental to advancing the country towards a sustainable and resilient future.

Academic researches on energy consumption structures are currently at the forefront due to the pressing global challenges in energy, environment, and economy [1]. Existing researches on energy mainly focused on energy efficiency [2], energy performance [3], and energy structure [4] to analyze their influencing factors, such as economic development [5], industrial structure [6], and urbanization [7]. However, there are relatively few studies on the energy consumption structure’s low-carbon transition process (Lct). The latest research on the Lct analyzed the synergistic effects of green credit and green technology innovation [8]. Regarding the researches on industrial agglomeration, existing researches focused on its impact on energy dynamics [9] and total factor energy efficiency [10]. It can be seen that existing researches had not explored the relationship between industrial agglomeration and the Lct, and is still in a blank stage. Regarding the measurement of the Lct, there are currently no unified research method in the academic community. Some scholars used a simple indicator system to measure it [11], and some scholars used the principal component analysis method (PCA) to construct an indicator system [12]. However, these methods are highly subjective and difficult to express the energy consumption structure accurately.

Given this, this paper uses the spatial vector angle method and only considers three types of energy: coal, oil, and natural gas to construct an indicator system to measure the Lct. At the same time, to fill the gap in existing researches on the relationship between industrial agglomeration and the Lct, this paper constructs a spatial Durbin model containing quadratic terms of industrial agglomeration to analyze the nonlinear and spatial spillover effects of industrial agglomeration. The novelty and marginal contribution of this article lie in the following aspects:

(1) This study particularly emphasizes the spatiotemporal evolution trends of industrial agglomeration and Lct, and also conducts regional heterogeneity analysis, thus significantly broadening the research horizons of industrial agglomeration and Lct.

(2) Considering the crowding effect of industrial agglomeration, this study innovatively incorporates the square term of industrial agglomeration into the model to analyze the nonlinear inverted U-shaped impact on Lct.

(3) This study innovatively adds the square term of industrial agglomeration to the dual-space Durbin model, revealing the nonlinear spatial spillover effect of industrial agglomeration on Lct.

(4) This paper uses the spatial vector angle method to measure the Lct and deal with the complex structural evolution of subdivided energy, which is comparable across samples.

This study enhances academic understanding of the relationship between industrial agglomeration and Lct. It also helps us better understand the complex interactions between industrial dynamics, environmental sustainability, and social well-being. This study has important reference significance for the industrial pattern distribution of various provinces in China and provides valuable insights for industrial stakeholders and policymakers. In addition to its implications for the industrial sector, this research has broader implications for broader social issues such as sustainable development, regional inequality, and environmental sustainability. These findings can help policymakers and stakeholders chart a more inclusive, resilient, and sustainable path to move China’s industry and society toward shared prosperity and sustainability.

## 2. Literature review

### 2.1 Industrial agglomeration and the inverted-U relationship

**Fig 1:**
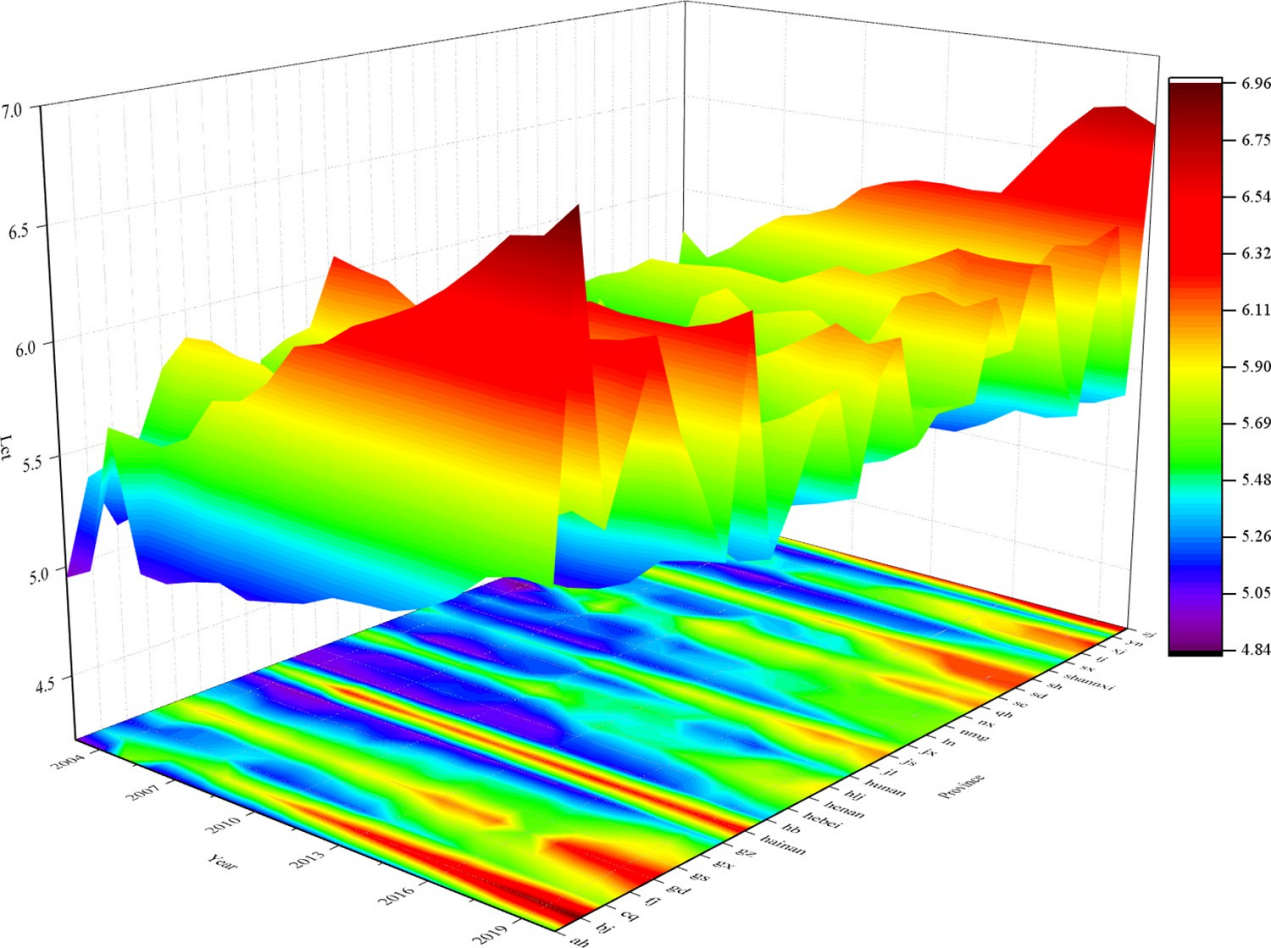
Lct’s 3D surface map.

The 3D surface and mapping of the spatiotemporal evolution trend of the Lct in 30 Chinese provinces from 2003 to 2020 are shown in Fig 1. Where the y-axis is the year, i.e., 2003–2020, the z-axis is the size of Lct, and the x-axis represents the chosen 30 provinces. The image shows that whereas central and western regions like Shaanxi, Shanxi, and Hubei have historically had lower degrees of Lct, provinces like Beijing, Fujian, Guangdong, and Zhejiang have gradually strengthened the degree of Lct through time.

The standard method for studying industrial agglomeration is based on a scalar measure of agglomeration within each industry. Mori and Smith (2015) proposed a quantitative method based on an explicit method of detecting spatial clusters to differentiate the scale and extent of industrial agglomeration [13]. They improved on their own to make it representative of spatial agglomeration. Shi et al. (2023) conducted a visual analysis of industrial agglomeration’s spatial distribution pattern for visualization and analysis [14], constructed the network of inter-city traffic interaction, and then inferred the network of population mobility trend and technological innovation mobility trend. Based on the regression analysis model considering the spatial intensity of mobility, the specific relationship between industrial agglomeration and the influencing factors was studied. There are two views on industrial agglomeration: the agglomeration effect and the congestion effect. The externality theory of industrial agglomeration represented by Lekachman (1962) believes that the positive economic externality is a distinctive feature of industrial agglomeration as a form of organization and takes this as an essential source to maintain the competitiveness of industrial agglomeration [15]. With the development of the theory, scholars further found that industrial agglomeration also has obvious energy-positive externalities, and from the collaborative innovation environment between enterprises related to the spillover of clean technology [16], the scale effect of pollution control and the circular economy and other perspectives, that industrial agglomeration organizational form, could promote economic growth, but also to accelerate the effect of energy structure transformation [17]. In addition, there are theories that the industrial agglomeration process would have negative externalities [18]. New economic geography [19] believed that industrial agglomeration, to a certain extent, might have a congestion effect, and the energy consumption structure was one of the specific manifestations of the above phenomenon. Zhang and Tao (2023) investigated the inverted U-shaped relationship between the green economy and industrial agglomeration in the Yangtze River Delta region [20]. The magnitude of agglomeration and crowding effects of industrial agglomeration could be illustrated by the output density model proposed by Ushifusa and Tomohara (2013) to explain the changes and constraints between them [21].

### 2.2 Spatial spillover effects of industrial agglomeration

In recent years, scholars had gradually studied the influence of industrial agglomeration on other factors from a spatial perspective. Cohen and Paul (2005) concluded that industrial agglomeration had a certain spillover effect at the spatial level [22]. Qin et al. (2022) studied the spatial convergence and correlation of industrial agglomeration on pollution emissions in the Yellow River Basin with the help of the spatial Durbin model [23]. They concluded that there were certain heterogeneous differences in the influence of industrial agglomeration on pollution emissions in the Yellow River Basin’s upper, middle, and lower reaches. Yang et al. (2023) used the location entropy model to calculate the collaborative industrial agglomeration [24], measured the nonlinear relationship between the collaborative industrial agglomeration and the atmospheric environmental efficiency based on the threshold regression model, and used the spatial Durbin model to study the spatial spillover effect of the joint industrial agglomeration on the atmospheric environmental efficiency effect. Xiong et al. (2022) used machine learning to process the multi-subject database and studied the spatial distribution characteristics of metal-related industrial agglomeration and the spatial-temporal differentiation characteristics of pollution emission, and finally put forward policy suggestions for sustainable development from the perspective of the spatial spillover effect of industrial agglomeration [25]. In summary, in the spatial impact of industrial agglomeration studies, most of them focused on the study of the spatial spillover effect of industrial agglomeration of urban agglomerations or industrial enterprise agglomeration on carbon emissions, environmental pollution, etc. There were few studies on industrial agglomeration and Lct. Whether industrial agglomeration has a significant positive impact on the Lct remains to be confirmed and is still in a blank stage.

### 2.3 Heterogeneity of industrial agglomeration

Yu et al. (2023) and others found that industrial agglomeration has spatial heterogeneity in sustainability [26]. It can be seen that it is necessary to discuss industrial agglomeration subregionally. From Fig 1, we can see that Lct is stronger in the eastern region. In contrast, the degree of Lct in the central and western regions is slightly inferior, so the regional differences formed by the economy, geography, and culture provide an opportunity for this paper to study the impact of different degrees of industrial agglomeration on the Lct. Therefore, this paper divides the 30 provinces into three regions: east, west, and central, and analyses the impact of industrial agglomeration on the Lct in different regions.

### 2.4 Spatial correlation test methodology

Compared to traditional econometrics, spatial econometrics considers the interaction between regions. It incorporates spatial effects into regression models through spatial weights, thereby enhancing the explanatory power and validity of the models [27]. Spatial effects primarily refer to spatial dependence and spatial heterogeneity. Spatial dependence implies that the attributes or behaviors of different regions are not independent but mutually influenced, with the degree of influence correlated with the absolute or relative positions of the regions. Spatial heterogeneity indicates significant regional differences in economic and social development due to regions’ varying economic and geographic characteristics [28].

Spatial correlation is captured by spatial weights, taking the spatial weight matrix of cross-sectional data as an example. Suppose there are n observational samples within the study area. The expression *W* is as follows.

$$W=(\begin{array}{ccc} w_{11} & \ldots & w_{1n}\\ \vdots & \ddots & \vdots \\ w_{n1} & \cdots & w_{nn} \end{array})$$ (1)

The elements *w*_*ij*_ in *W* define the spatial adjacency relationship of spatial objects. According to different adjacency criteria, when region *i* is adjacent to region *j*, *w*_*ij*_ = 1; when region *i* is not adjacent to region *j*, *w*_*ij*_ = 0. This study chooses the most commonly used R-neighborhood calculation for computing spatial weight values in the adjacency matrix. Compared to cross-sectional spatial weight matrices, panel spatial matrices incorporate a time factor (also known as time-varying spatial weight matrix). The spatial weight matrix constructed in this study is based on the adjacency among 30 major provinces and cities in China, excluding Hong Kong, Macau, Taiwan, and Tibet regions.

Typically, spatial correlation is measured using the global *Moran*’*s I*, while the detailed spatial distribution characteristics within the study object can be further examined using local *Moran*’*s I*. The formula for computing global *Moran*’*s I* is as follows:

$$Moran's\;I=\frac{\sum_{i=1}^{n}\sum_{j=1}^{n}w_{ij}(x_{i}-\bar{x})(x_{j}-\bar{x})}{S^{2}\sum_{i=1}^{n}\sum_{j=1}^{n}w_{ij}}$$ (2)

Where n is 30 provinces, cities, and autonomous regions, *w*_*ij*_ is the spatial weight. In this paper, the normalized spatial adjacency matrix is selected as the spatial weight matrix, x and $\bar{x}$ is the industrial agglomeration or the decarbonization transition of the energy consumption structure in each province, and its mean value *S*^2^ represents sample variance. *Moran*’*s I* is usually between -1 and 1, with greater than 0 representing positive spatial correlation and converging to 0 indicating no spatial correlation.

The formula for computing local Moran’s I is:

$$Moran's\;I=\sum w_{ij}z_{i}z_{j}$$ (3)

Where *z*_*i*_ and *z*_*j*_ are standardized observation values *w*_*ij*_ is the standardized spatial weight matrix. When *Moran*’*s I*_*i*_>0 and *z*_*i*_>0, the study variable exhibits high-high clustering, distributed in the first quadrant of Moran scatter plot; when *Moran*’*s I*_*i*_<0and *z*_*i*_<0, the study variable exhibits low-low clustering, distributed in the third quadrant of Moran scatter plot; when *Moran*’*s I*_*i*_<0 and *z*_*i*_>0, the study variable exhibits high-low clustering, distributed in the second quadrant of Moran scatter plot; when *Moran*’*s I*_*i*_>0 and *z*_*i*_<0, the study variable exhibits low-high clustering, distributed in the fourth quadrant of Moran scatter plot.

Based on this, the following hypotheses are proposed in this paper:

***H1***: The impact of industrial agglomeration on the Lct is the result of a combination of positive and negative aspects. When lower than a certain threshold, the agglomeration effect is greater than the crowding effect, which will accelerate the energy consumption structure’s low-carbon transition process; when higher than the threshold, the agglomeration effect will be smaller than the crowding effect, which will slow down Lct.
***H2***: The impact of industrial agglomeration on the Lct has spatial spillover effects.
***H3***: Heterogeneous industrial agglomerations have different impacts on the Lct.

## 3. Methodology and data

### 3.1 Model construction

This paper proposes to use a spatial econometric model to analyze industrial agglomeration and Lct.

#### 3.1.1 Spatial correlation tests

The first step in spatial measurement is a spatial autocorrelation test, and the commonly used index is the *Moran*’*s I*.

In this paper, the test of global spatial autocorrelation was carried out for the independent and dependent variables, respectively, and the results are shown in Table 1 below. We can see that Moran’s *I* of industrial agglomeration and the Lct in each year is greater than 0, and they are significant in the 99% confidence interval.

**Table 1:** Global Moran index values of 2003–2020.

Year	IA				Lct			
	I	Sd (I)	z	p-value	I	Sd (I)	z	p-value
2003	0.333	0.090	4.103	0.000	0.340	0.125	3.003	0.003
2004	0.330	0.092	3.964	0.000	0.339	0.124	3.023	0.003
2005	0.326	0.092	3.940	0.000	0.352	0.124	3.115	0.002
2006	0.324	0.092	3.908	0.000	0.376	0.121	3.397	0.001
2007	0.282	0.085	3.707	0.000	0.332	0.122	2.996	0.003
2008	0.278	0.085	3.652	0.000	0.343	0.121	3.114	0.002
2009	0.278	0.086	3.622	0.000	0.326	0.122	2.952	0.003
2010	0.277	0.086	3.613	0.000	0.316	0.123	2.841	0.004
2011	0.278	0.087	3.586	0.000	0.278	0.123	2.540	0.011
2012	0.280	0.088	3.553	0.000	0.294	0.123	2.681	0.007
2013	0.221	0.078	3.258	0.001	0.339	0.123	3.042	0.002
2014	0.224	0.080	3.244	0.001	0.332	0.123	2.983	0.003
2015	0.228	0.081	3.247	0.001	0.322	0.123	2.906	0.004
2016	0.230	0.082	3.245	0.001	0.225	0.122	2.132	0.033
2017	0.231	0.082	3.246	0.001	0.275	0.122	2.531	0.011
2018	0.232	0.082	3.266	0.001	0.247	0.122	2.312	0.021
2019	0.236	0.082	3.282	0.001	0.197	0.122	1.906	0.057
2020	0.221	0.077	3.322	0.001	0.222	0.121	2.112	0.035

**Fig 2:**
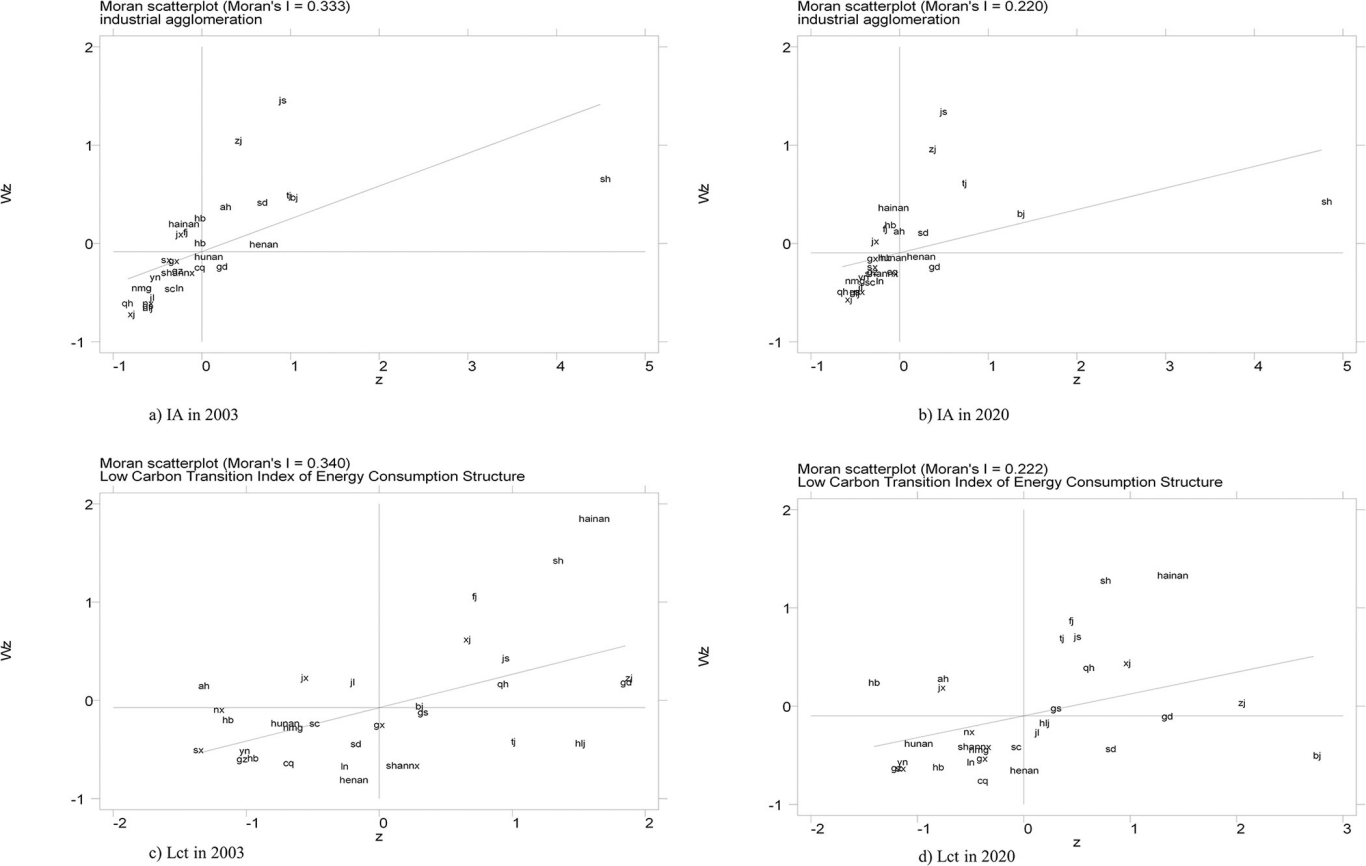
Localized Moran index chart.

Fig 2 shows the localized *Moran*’*s I* results. From a) and b), it can be seen that the local Moran index of industrial agglomeration in 2003 and 2020 are 0.333 and 0.220, respectively, which show strong local positive spatial correlation; c) and d) in Fig 2 are the local Moran index of the Lct in 2003 and 2020, respectively, which are greater than 0, and there exists strong local positive spatial autocorrelation.

**3.1.2 Spatial modeling tests.** Given that it considers various spatial interactions as opposed to the typical panel model, the spatial panel econometric model is better appropriate for the situation of this paper. The following forms of the spatial lag model (SAR), spatial error model (SEM), and spatial Durbin model (SDM) are the most often used spatial econometric models. The spatial Durbin model (SDM), which incorporates the spatial lag terms of the explanatory factors and the explanatory variables, is one of them. It is an enhanced version of the SAR and SEM.

$$\left\{ \begin{array}{l} SAR:\;Y=\delta WY+X\beta +\varepsilon \\ SEM:Y=WX\theta +X\beta +\mu ,and\;\mu =\lambda W\mu +\varepsilon \\ SDM:Y=\delta WY+X\beta +WX\theta +\varepsilon \end{array}\right.$$ (4)

In the above equation, *Y* represents the dependent variable matrix; W represents the spatial weight matrix; and *WY* represents the spatial lag term of the dependent variable; *δ* represents the spatial autoregressive coefficient, which is used to indicate the degree of interaction between areas; *X* represents the matrix of explanatory variables; *β* represents the corresponding coefficient variable; *WX* represents the spatial lag term of the explanatory variables; *θ* is the corresponding coefficient vector。*ε* is a random perturbation term, *λ* is the error space autoregression coefficient, *μ* is the vector of regression residuals.

According to Elhorst (2014), a Lagrange multiplier test is necessary to select spatial econometric models [29]. Table 2 shows that both lag and error multipliers passed the test at a 1% confidence level, demonstrating spatial effects. Additionally, the robustness error multiplier is considerable at the 1% level, demonstrating the use of SEM. As indicated in Table 2 below, the LR and Wald test results passed the test at a 1% confidence level, rejecting the initial hypothesis. However, it is still essential to conduct the LR and Wald tests to decide whether SDM will degenerate into SEM or SAR. In conclusion, this work can utilize the SDM model.

**Table 2:** Spatial econometric modeling tests.

Test Methods	statistical value	p-value
LM-lag	51.323***	0.000
Robust LM-lag	70.816***	0.000
LM-error	132.497***	0.0000
Robust LM-error	151.990***	0.0000
Wald Spatial error	59.10***	0.0000
Wald Spatial lag	62.17***	0.0000
LR Spatial error	56.13***	0.0000
LR Spatial lag	58.65***	0.0000
Hausman	18.42***	0.0183

Notes: Values in parentheses indicate the standard deviation.

*, ** and *** indicate p < 0.1, p < 0.05 and p < 0.01, respectively.

Additionally, the paper conducted the Hausman test, and the results rejected the original hypothesis. So, this paper should use the Spatial Durbin Model (SDM) with double fixed effects.

### 3.2 Variable selection

#### 3.2.1 The explanatory variables

The energy consumption structure’s low-carbon transition process (Lct). The systematic project of continuously improving and changing the numerous dominating energy sources in complementing and substituting is known as the Lct. To speed up the clean-up of energy consumption, it primarily refers to the trend of replacing high-carbon characteristics with green and low-carbon features in China’s energy structure. In this study, we developed the Lct based on the spatial vector angle method [30] to assess the Lct.

First, oil and gas, coal, and other energy consumption are the three primary energy sources consumed. The consumption share of each type of energy in the year(t) is a component of a spatial vector, which can form a set of 3-dimensional vectors $L_{t}({l_{t}}^{1},{l_{t}}^{1},{l_{t}}^{1})$。

Secondly, calculate the angle ${\theta_{t}}^{1}、{\theta_{t}}^{2}、{\theta_{t}}^{3}$ of *L*_*t*_ and the energy consumption vector ranked from high to low carbon ${L_{0}}^{1}=(1,0,0),{L_{0}}^{2}=(0,1,0),{L_{0}}^{3}=(0,0,1)$.


$${\theta_{t}}^{i}=\arccos\{\frac{\sum_{j=1}^{3}\left({l_{t}}^{j}\times {l_{0}}^{j}\right)}{\left[\sum_{j=1}^{3}{\left({l_{t}}^{j}\right)}^{2}\times \sum_{j=1}^{3}{\left({l_{0}}^{j}\right)}^{2}\right]}\}i=1,2,3$$ (5)

Thirdly, a weighted average of all vectors for each year t is used to obtain the energy consumption structure decarbonization index *Lct*_*t*_, calculated as follows:

$$Lct_{t}=\sum_{s=1}^{3}\sum_{t=1}^{s}\theta_{t}^{j}$$ (6)

**Fig 3:**
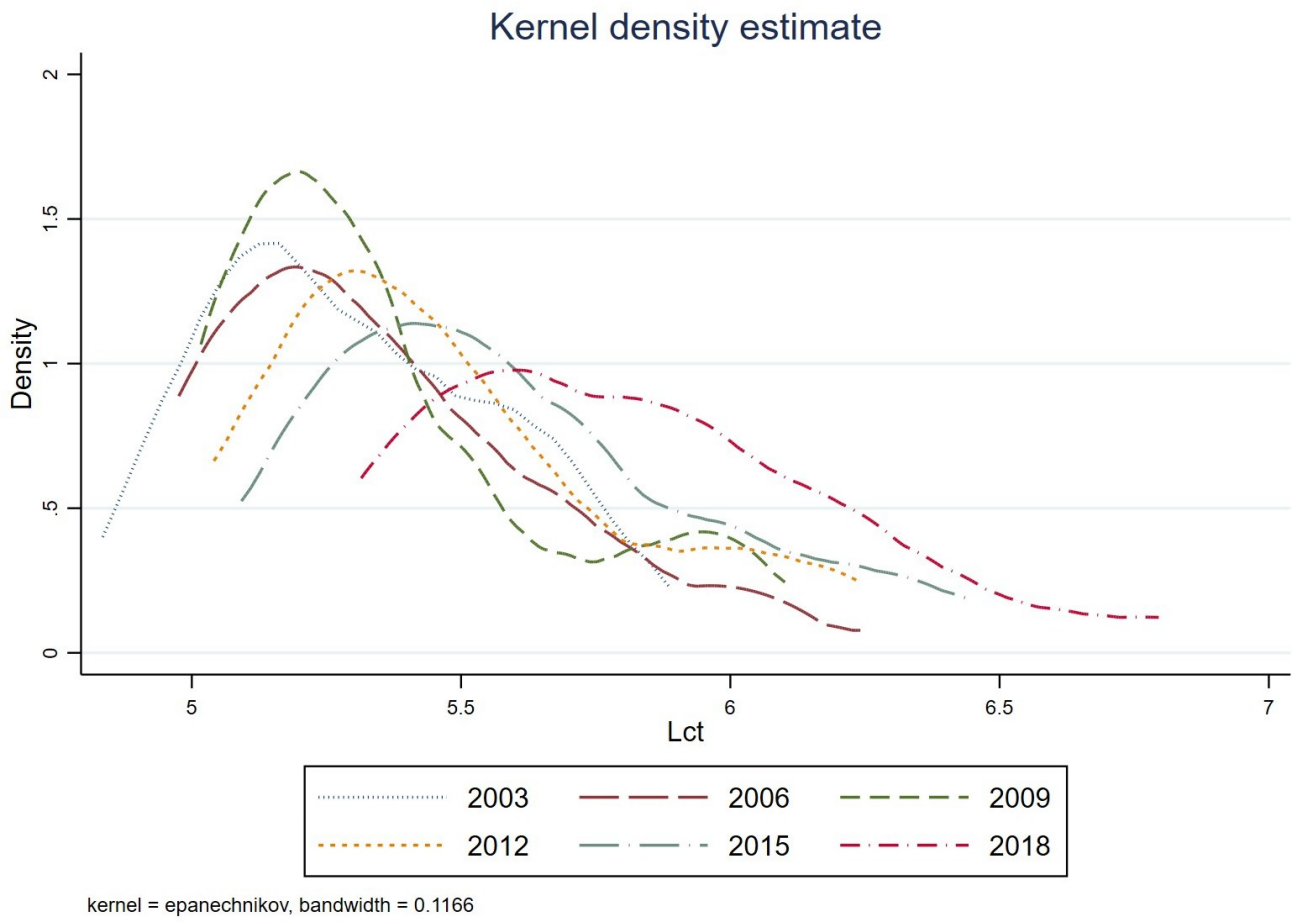
Kernel density of Lct from 2003 to 2020.

To analyze the differences and dynamic changes of Lct during different periods, this article presents the kernel density of Lct for different years, as shown in Fig 3. It shows that the peak of the nuclear density curve for energy efficiency gradually increased from 2003 to 2020, indicating that Lct continues to improve during the sample period.

#### 3.2.2 Core explanatory variables

Industrial agglomeration (IA). The term "industrial agglomeration" describes combining different subjects, such as the government, businesses, universities, and research institutions, who interact through interdependence, cooperation, complementary strengths, improved labor division, resource sharing, etc., to foster collaborative innovation. In this study, the employment density of each region was chosen to represent the level of industrial agglomeration; the higher the population density, the higher the level of industrial agglomeration.

#### 3.2.3 Control variables

In light of studies by Chang et al. [26, 31] and others [10], we chose the following control variables. The ratio of the urban to the overall population is known as the urbanization level (civil). The ratio of industrial value added to GDP is known as the industrialization level (gysp). The ratio of foreign direct investment to the gross regional product is known as foreign direct investment (fdi). The proportion of total retail sales of consumer goods to GDP is known as the social consumption level (sxsp). The logarithm of total freight traffic is known as the Transport Infrastructure Level (jtsp). The ratio of completed investments in industrial pollution control to industrial value added is known as environmental regulation (envir). The proportion of fiscal expenditure to GDP indicates the degree of government interference (gov). The aforementioned factors are specified as the controls influencing the Lct.

### 3.3 Data

This study chose panel data from 30 Chinese provinces (municipalities and autonomous areas) spanning 18 years from 2003 to 2020 for analysis because of the severe absence of data in Tibet, Hong Kong, Macao, and Taiwan. *The China Urban Statistical Yearbook*, *EPS*, *CSMAR*, *province statistical yearbooks*, and *CNRDS* are the main sources of the pertinent data, and the average growth rate approach and interpolation are used to fill in the gaps. Table 3 displays the descriptive statistics for each variable.

**Table 3:** Descriptive statistics.

VARIABLES	N	mean	sd	min	max
Lct	540	5.522	0.381	4.846	6.956
IA	540	0.0244	0.0343	0	0.217
cjcbz	540	0.0522	1.064	-0.693	6.030
cival	540	0.533	0.148	0.139	0.896
gysp	540	0.343	0.0861	0.101	0.559
fdi	540	0.0232	0.0191	0.000100	0.105
sxsp	540	0.127	0.110	0.00365	0.644
jtsp	540	11.21	0.917	8.526	12.94
envir	540	0.00419	0.00363	8.50e-05	0.0279
gov	540	0.207	0.0947	0.0772	0.643

## 4. Empirical results and discussion

### 4.1 Unit root and cointegration tests for spatial panels

Considering the common trends in the data, there may be pseudo-regression problems. So, to avoid pseudo-regression and to ensure the validity and unbiasedness of the results, a stationarity test was carried out before fitting the model [32]. This paper used the HT and ADF tests in unit root tests, and the test results are shown below. It can be seen that most of the variable series are non-stationary at the 10% level. After that, this paper repeatedly verified the series after first-order differencing of all variables, and it can be obtained that they are all smooth series.

From the smoothness test, it can be seen that the logarithmic values of the above variables satisfy the first-order simple integer. Next, this paper needs to examine whether there is a cointegration relationship between the selected variables. Considering the time trend of the sample, this paper selected the Pedroni test for the cointegration test [33]. The three test statistics of Pedroni are reported in Table 4 below have a p-value of 0.0000, indicating that the original hypothesis of "no cointegration" is strongly rejected, i.e., a cointegration relationship exists. Therefore, this paper can use the original series for regression analysis.

**Table 4:** Stationarity test result of variable.

	HT	ADF-Fisher	Steady or not
Gec	0.0003	0.0000	Yes
Cycbz	0.6808	1.0000	No
Cival	0.0002	0.0000	Yes
Gov	0.9990	0.2111	No
Envir	0.0000	0.3861	No
Fdi	0.4991	0.0971	No
Sxsp	0.7783	0.7073	No
Jtsp	0.9998	1.0000	No
Gysp	1.0000	0.9610	No
Pedroni test for cointegration	Statistic	p-value	Cointegration or not
Modified Phillips–Perron t	7.0570	0.0000	Yes
Phillips–Perron t	-11.4702	0.0000	Yes
Augmented Dickey–Fuller t	-8.6228	0.0000	Yes

### 4.2 Regression results

The M1-M4 model is as follows:

$$\left\{ \begin{array}{l} OLS:\;Lct=\alpha_{0}+\alpha_{1}IA+\alpha_{2}IA^{2}+\alpha_{i}Controls+\mu_{i}+\phi_{t}+\varepsilon_{i}\\ SAR:Lct=\gamma W\times Lct+\eta_{1}IA+\eta_{2}IA^{2}+\eta_{3}Controls+\mu_{i}+\phi_{t}+\varepsilon_{i}\\ SEM:Lct=W\times IA+W\times IA^{2}+\gamma_{1}IA+\gamma_{2}IA^{2}+\gamma_{3}Controls+\mu_{i}+\phi_{t}+\zeta \\ SDM:Lct=\delta_{1}W\times Lct+\delta_{2}W\times IA+\delta_{3}W\times IA^{2}+\delta_{4}W\times Controls+\varphi_{1}IA+\varphi_{2}IA^{2}+\varphi_{3}Controls+\mu_{i}+\phi_{t}+\varepsilon_{i} \end{array}\right.$$ (7)

M1–M4 in Table 5 provides the findings of the OLS, SEM, SAR, and SEM model regressions. All spatial econometric models are estimated using the method of maximum likelihood estimation (MLE), and the spatial matrices used in the models are all 0–1 adjacency matrices. Doing this can eliminate some endogeneity issues. The coefficients of industrial agglomeration on the Lct are 16.221, 7.340,5.242, and 13.597, which pass the 1% significance level test and are all positive, as shown in Table 5 when industrial agglomeration is the primary explanatory variable. However, the squared term’s coefficients are all negative and significant at the 1% level. This demonstrates that industrial agglomeration significantly alters the structure of low-carbon energy use in an inverted-U relationship. Based on prior research, it is known that early industrial agglomeration will result in an expansion of the market scale, which will result in the phenomena of labor sharing, intermediate input product sharing, and knowledge spillover. To a certain extent, the scale effect will predominate, encouraging the Lct. Industrial agglomeration, however, will harm the Lct after it has developed to a certain extent. This may be because the rapid expansion of production capacity within the industrial clusters has sharply increased the energy demand, and the speed of energy structure transformation cannot keep up with the increase in the energy demand rate, which causes it to slow down. This is congruent with the findings of the paper’s theoretical derivation section. ***H1*** is confirmed.

**Table 5:** Estimation results.

	M1	M2	M3	M4	
	OLS	SEM	SAR	SDM	
main					Wx
IA	16.221***	7.340***	5.242***	13.597***	94.996***
	(2.806)	(1.427)	(1.212)	(2.610)	(16.267)
IA^2^	-44.320***	-37.065***	-27.089***	-33.510***	-181.061***
	(8.329)	(6.455)	(5.593)	(7.622)	(46.175)
cival	-0.164	0.818***	0.768***	0.041	0.524
	(0.121)	(0.133)	(0.127)	(0.108)	(0.715)
gov	-0.104	0.501**	0.610***	-0.115	-2.715***
	(0.186)	(0.231)	(0.219)	(0.184)	(0.973)
envir	-4.041*	-12.452***	-13.572***	-2.853	-1.726
	(2.303)	(3.455)	(3.402)	(2.056)	(14.947)
fdi	-1.049*	2.025**	1.911**	0.185	-3.028
	(0.636)	(0.860)	(0.861)	(0.595)	(3.836)
sxsp	0.013	-0.711***	-0.745***	-0.102	-0.015
	(0.108)	(0.118)	(0.116)	(0.102)	(0.723)
jtsp	0.019	-0.045**	-0.050**	0.002	-0.421***
	(0.023)	(0.022)	(0.022)	(0.021)	(0.107)
gysp	0.247	-0.625***	-0.355**	0.122	0.242
	(0.181)	(0.167)	(0.159)	(0.164)	(0.987)
_cons	5.057***				
	(0.289)				
Spatial		0.398***	0.339***	0.238*** (0.058)	
rho		(0.058)	(0.050)		
N	540.000	540.000	540.000	540.000 0.461	
R^2^	0.906	0.460	0.496		

Notes: Values in parentheses indicate the standard deviation.

*, ** and *** indicate p < 0.1, p < 0.05 and p < 0.01, respectively.

The linear term and quadratic term of the industrial agglomeration’s spatial spillover term, presented in M4, have positive and negative coefficients, respectively, and are significant at the 1% confidence level. This shows that the shift to the Lct also exhibits a large inverted-U relationship in the regional spillover impact of industrial agglomeration. Industrial clusters can aid in creating a platform for green energy technology innovation and sharing among industries in the early stages of industrial agglomeration [34]. They can also encourage the sharing of advanced technology and management experience in energy use upgrading, which can inspire and teach the neighboring industrial clusters, accelerating the spread of the Lct [35]. Although excessive agglomeration causes the competitive relationships between industries to intensify and extrude one another, the agglomeration effect of industry is transformed into a crowding effect [36] in the late stages of industrial agglomeration. As a result, the spatial effect of industrial agglomeration may have some unfavorable effects on other provinces.

### 4.3. Effect decomposition

The equation for effect decomposition is as follows:

$$Directeffect={\left[{\left(I-\rho W\right)}^{-1}(\alpha_{k}I)\right]}^{\bar{d}}$$ (8)

$$Indirecteffect={\left[{\left(I-\rho W\right)}^{-1}(\alpha_{k}I)\right]}^{rs\bar{u}m}$$ (9)

This paper uses effect decomposition [29] to estimate the direct, indirect, and total effects (Arnold, 2011) to analyze the intrinsic mechanism of industrial agglomeration’s impact on the shift to the Lct because the results of the above regression cannot specifically reflect the marginal impact of the independent variables on the dependent variable. Table 6 illustrates the three effects of the linear and quadratic terms of industrial agglomeration, which are positive and negative at the 1% level. The validity of the hypothesis H2 can be deduced. When the indirect impacts are further examined, it becomes clear that the spatial impact of industrial agglomeration is the primary determining element because the absolute values of all the indirect effects are substantially higher than the direct effects. This suggests that the transition process toward a low-carbonization of the energy consumption structure in neighboring provinces is significantly influenced by the substantial externality feature of industrial agglomeration. This supports the fundamental premise ***H2*** of the earlier paper.

**Table 6:** Decomposition of effects in the spatial Durbin model.

	Direct effect	Indirect effect	Total effect
IA	10.018***	37.209***	47.227***
	(3.027)	(8.787)	(7.227)
IA2	-27.457***	-65.850***	-93.307***
	(8.450)	(23.494)	(19.915)
cival	0.031	0.224	0.255
	(0.122)	(0.377)	(0.359)
gov	0.024	-1.305***	-1.282***
	(0.231)	(0.473)	(0.355)
envir	-3.447	1.863	-1.584
	(2.509)	(6.698)	(6.346)
fdi	0.371	-1.633	-1.262
	(0.603)	(1.831)	(1.766)
sxsp	-0.112	0.066	-0.047
	(0.099)	(0.340)	(0.329)
jtsp	0.018	-0.198***	-0.180***
	(0.020)	(0.050)	(0.046)
gysp	0.099	0.072	0.171
	(0.157)	(0.447)	(0.458)

Notes: Values in parentheses indicate the standard deviation.

*, ** and *** indicate p < 0.1, p < 0.05 and p < 0.01, respectively.

### 4.4 Robustness tests

The spatial econometric model is then tested for robustness to confirm the validity of the empirical findings in this work. First, replacing the explanatory factors. The normalized industrial agglomeration index is used in this study to regress the Lct, as shown in M5. The linear term and quadratic term coefficients are consistent with the trend above, and both are significant at the 1% level, indicating that the model is reliable. Secondly, lagging one-period explanatory variables. The model is reliable because LIA_1 and LIA_2, shown in M6 as the linear term and quadratic term of one lag, respectively, and their regressed coefficients, are in the same direction and have the same significance as above. Finally, reducing the first and last outliers. The model is reliable because, as shown in M7, the outliers of the independent variable and the dependent variable are 99% winsorized, and the final findings in Table 7 show that the primary term and the quadratic term’s coefficients’ direction and significance are unaltered.

**Table 7:** Robustness test results.

	M5 Replacing explanatory variables		M6 Explanatory variables lagged		M7 outlier reduction	

Main		Wx		Wx		Wx
IAS	0.390***	2.956***				
	(0.075)	(0.471)				
IAS2	-0.035***	-0.203***				
	(0.008)	(0.049)				
cival	0.047	0.540	0.084	1.051	0.092	0.745
	(0.108)	(0.713)	(0.105)	(0.692)	(0.105)	(0.694)
gov	-0.122	-2.650***	-0.132	-2.423**	-0.125	-2.313**
	(0.183)	(0.970)	(0.181)	(0.952)	(0.179)	(0.946)
envir	-2.884	-2.140	-2.106	6.292	-2.922	-2.997
	(2.049)	(14.898)	(2.013)	(14.778)	(1.999)	(14.552)
fdi	0.175	-3.264	0.355	0.807	0.273	0.073
	(0.592)	(3.814)	(0.586)	(3.820)	(0.579)	(3.731)
sxsp	-0.104	-0.028	-0.125	-0.521	-0.119	-0.394
	(0.102)	(0.719)	(0.100)	(0.718)	(0.100)	(0.702)
jtsp	0.001	-0.420***	0.005	-0.387***	0.004	-0.418***
	(0.021)	(0.106)	(0.021)	(0.104)	(0.021)	(0.104)
gysp	0.139	0.427	0.147	0.188	0.098	-0.347
	(0.164)	(0.988)	(0.161)	(0.950)	(0.160)	(0.958)
LIA_1			11.377***	96.654***		
			(2.476)	(15.003)		
LIA_2			-26.702***	-181.711***		
			(7.348)	(43.123)		
IA					11.620***	97.045***
					(2.552)	(15.724)
IA2					-27.710***	-182.862***
					(7.489)	(45.076)
					(45.076)	
Spatial						
rho	-1.303***		-1.391***		-1.251***	
	(0.191)		(0.191)		(0.192)	
N	540.000		540.000		540.000	
R^2^	0.174		0.189		0.181	

Notes: Values in parentheses indicate the standard deviation.

*, ** and *** indicate p < 0.1, p < 0.05 and p < 0.01, respectively.

## 5. Heterogeneity test

The economics, culture, and customs of each province in China vary somewhat because of the country’s colossal landmass [37]. As a result, 30 provinces are separated into three areas in this study for the sake of regression analysis: east, middle, and west.

**Fig 4:**
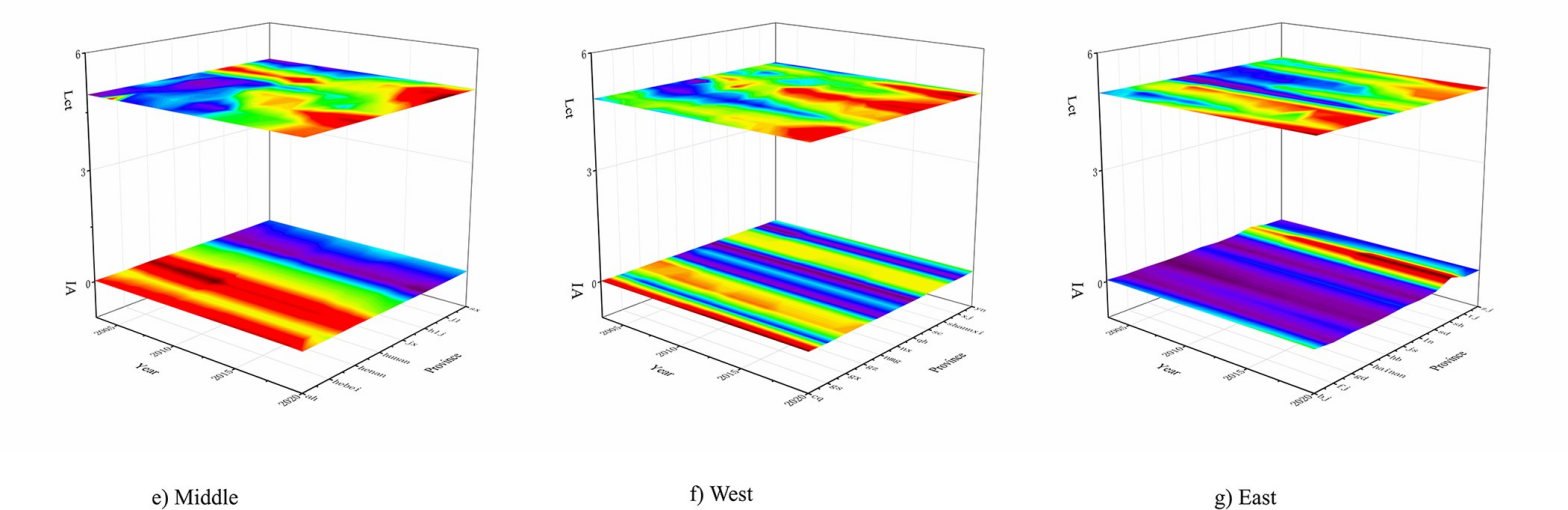
Regional 3D stacked map. (e) 3D stacked map of the central region; (f) 3D stacked map of the western region; (g) 3D stacked map of the eastern region.

According to the degree of industrial agglomeration and the Lct in the east, west, and center zones, Fig 4 is a three-bit stacked diagram. The plane on top of Fig 4 represents the mapping map for the decarbonization transition of the energy consumption structure of Lct. The plane on the bottom of Fig 4 represents the mapping map for the degree of industrial agglomeration of industrial agglomeration, where the x-axis indicates the province of the region to which it belongs, and the y-axis indicates the year. The z-axis indicates the size of the degree of industrial agglomeration and the Lct. To more effectively compare the size and distribution of the independent and dependent variables across different time and space, the two stacked planes in this study are projected in three dimensions. It improves the results’ intuitiveness.

Table 8 demonstrates that only the eastern region experiences the inverted-U relationship of industrial agglomeration on the Lct; in contrast, the central and western regions do not experience any discernible effects. This demonstrates that the gap in industrial agglomeration between the regions has grown, and the developed eastern region continues to acquire more resources, creating a siphon effect [38]. This is due to the more developed economy in the eastern region, the high degree of concentration of universities and research institutes, and the high degree of industrial agglomeration. Due to their inferior economic development, lack of resources, unfavorable environmental conditions, and lack of infrastructure development, the central and western areas cannot match the effect of industrial agglomeration with the eastern region. ***H3*** is confirmed.

**Table 8:** Regional heterogeneity regression results.

	(1)		(2)		(3)	
	East		Middle		West	
Main		Wx		Wx		Wx
IA	22.098***	85.650***	23.049	184.389	-4.836	-111.336
	(1.841)	(7.492)	(52.354)	(237.711)	(83.217)	(330.574)
IA^2^	-74.320***	-297.184***	-44.057	-2892.566	151.911	2597.877
	(6.940)	(29.412)	(2014.364)	(8068.239)	(1564.775)	(6084.472)
cival	-1.035***	-3.049***	0.677	6.136	1.144	8.986
	(0.199)	(0.620)	(0.717)	(5.297)	(3.015)	(10.654)
gov	-1.051***	0.165	-1.551***	-5.415***	2.226**	8.366**
	(0.273)	(1.156)	(0.327)	(1.614)	(0.943)	(3.737)
envir	3.860**	-10.958	-0.075	18.573	-6.695	-35.737*
	(1.744)	(7.224)	(2.408)	(13.642)	(6.052)	(21.037)
fdi	-1.880***	-1.797	-0.197	-2.134*	-0.166	0.709
	(0.452)	(1.745)	(0.190)	(1.189)	(0.386)	(1.383)
sxsp	0.047	0.067	-0.043	-0.234	-0.427***	-1.702***
	(0.039)	(0.154)	(0.041)	(0.217)	(0.083)	(0.301)
jtsp	-0.916	-5.500**	-0.069	2.099	-1.669	-10.298*
	(0.586)	(2.255)	(0.256)	(1.889)	(1.447)	(5.295)
gysp	-27.692***	-39.557	-3.438	-14.042	5.885	46.148
	(7.193)	(28.416)	(2.592)	(14.859)	(14.898)	(55.744)
Spatial						
rho	-0.469***		-1.088***		-1.040***	
	(0.152)		(0.231)		(0.201)	
N	234.000		198.000		108.000	
R^2^	0.453		0.378		0.050	

Notes: Values in parentheses indicate the standard deviation.

*, ** and *** indicate p < 0.1, p < 0.05 and p < 0.01, respectively.

## 6. Conclusion

Accurately understanding the impacts of industrial agglomeration on energy consumption structure’s low-carbon transition process is crucial in promoting high-quality development of industry and socio-economic progress. Based on panel data from 30 provinces in China spanning the years 2003 to 2020, this study utilized the spatial vector angle method to measure indicators of the energy consumption structure’s low-carbon transition process. Additionally, a nonlinear spatial Durbin model was constructed to explore industrial agglomeration’s nonlinear spatial spillover effects on the energy consumption structure’s low-carbon transition process. The study’s findings reveal several vital insights:

(1) Temporal and spatial evolution trends. Firstly, from Figs 1 and 3, we find that from 2003 to 2020, the degree of Lct has steadily increased annually. Spatially, the coastal regions in the eastern part of China exhibit a higher degree of Lct, with cities such as Beijing, Fujian, Guangdong, Shandong, Shanghai, and Hainan showing notable progress. Secondly, from the Fig 2’s local Moran’s I map, we found that industrial agglomeration demonstrates a significant positive spatial correlation, with high-high agglomeration primarily concentrated in Tianjin, Beijing, Anhui, Henan, and Shandong. In contrast, low-low agglomeration is mainly found in Inner Mongolia, Qinghai, Gansu, Shaanxi, and Shanxi. Similarly, the Lct exhibits significant positive spatial correlation, with high-high agglomeration primarily concentrated in Jiangsu, Fujian, and Shanghai, and low-low agglomeration mainly concentrated in Shaanxi, Inner Mongolia, and Ningxia. Thirdly, from the heterogeneity test, we found that industrial agglomeration significantly impacts the Lct in the eastern region. In contrast, the impact is insignificant in the central and western regions. Through all the analyses above, it can be inferred that while China’s industrial agglomeration and the transformation of energy consumption structures have shown steady long-term growth, spatial disparities and inequalities persist.

(2) Nonlinear effects: Industrial agglomeration significantly expedites the shift towards the energy consumption structure’s low-carbon transition process, aligning closely with the model’s foundational assumptions. A pronounced inverted-U relationship exists between industrial agglomeration and the energy consumption structure’s low-carbon transition process.

(3) Spatial Spillover Effects: Spatial spillover effects stemming from industrial agglomeration extend their impact to nearby areas. These effects are nonlinear, demonstrating a robust inverted-U relationship.

Based on the findings above, this research proposes the following recommendations:

(1) Given the steady annual increase in Lct degree and the spatial concentration of progress in coastal regions, policymakers should prioritize further investment and support in these areas to sustain and potentially accelerate this positive trend. Additionally, targeted interventions should be devised to address spatial disparities, particularly in regions with lower Lct levels, to promote more equitable development across the country [39].

(2) Recognizing the significant role of industrial agglomeration in expediting the shift towards low-carbon energy consumption structures, policymakers should leverage this relationship to facilitate a more rapid decarbonization transition. Strategies could include incentivizing clean energy investments and promoting technological innovation within agglomerated industrial zones. Furthermore, policies should be implemented to ensure the transition remains sustainable, avoiding potential negative impacts of the nonlinear relationship. Provincial governments are advised to manage the scale of industrial agglomeration judiciously, paying close attention to preventing congestion effects from excessive agglomeration [40]. Rejecting indiscriminate industry clustering while leveraging industrial agglomeration to expedite the energy consumption structure’s decarbonization transition is crucial [41].

(3) Collaborative regional development strategies should be devised to maximize the positive spatial spillover effects of industrial agglomeration on neighboring areas. This may involve fostering synergies between agglomerated regions and their adjacent areas through coordinated infrastructure development, knowledge exchange, and innovation networks. Additionally, policies should be designed to mitigate potential congestion effects and ensure that the benefits of industrial agglomeration are distributed more evenly across regions [42].
